# Monoclonal Antibodies Radiolabeling with Rhenium-188 for Radioimmunotherapy

**DOI:** 10.1155/2017/5923609

**Published:** 2017-08-30

**Authors:** Licia Uccelli, Petra Martini, Micol Pasquali, Alessandra Boschi

**Affiliations:** ^1^Morphology, Surgery and Experimental Medicine Department, University of Ferrara, Via L. Borsari 46, 44122 Ferrara, Italy; ^2^Physics and Heart Science Department, University of Ferrara, Via Giuseppe Saragat, 1, 44122 Ferrara, Italy; ^3^Legnaro National Laboratories, Italian National Institute for Nuclear Physics (LNL-INFN), Viale dell'Università 2, Legnaro, 35020 Padua, Italy

## Abstract

Rhenium-188, obtained from an alumina-based tungsten-188/rhenium-188 generator, is actually considered a useful candidate for labeling biomolecules such as antibodies, antibody fragments, peptides, and DNAs for radiotherapy. There is a widespread interest in the availability of labeling procedures that allow obtaining ^188^Re-labeled radiopharmaceuticals for various therapeutic applications, in particular for the rhenium attachment to tumor-specific monoclonal antibodies (Mo)Abs for immunotherapy. Different approaches have been developed in order to obtain ^188^Re-radioimmunoconjugates in high radiochemical purity starting from the generator eluted [^188^Re]ReO_4_^−^. The aim of this paper is to provide a short overview on ^188^Re-labeled (Mo)Abs, focusing in particular on the radiolabeling methods, quality control of radioimmunoconjugates, and their in vitro stability for radioimmunotherapy (RIT), with particular reference to the most important contributions published in literature in this topic.

## 1. Introduction

Radioimmunotherapy (RIT) represents a selective internal radiation therapy by means of *β*- or *α*-emitting radionuclides conjugated to tumor-directed (Mo)Abs, fragments, or peptides [1]. A selected radiolabeled (Mo)Abs, including fragment, can deliver high therapeutic radiation dose to cancer cells while minimizing the exposure of normal cells by selective interaction to cancer-associated antigens on tumor cell surface [2, 3]. Some examples of radiolabeled antibodies for RIT are ^90^Y-murine anti-CD20 antibody; ibritumomab, approved for clinical practice for the treatment of lymphoma some years ago [4]; ^131^I-tositumomab, which recognizes and binds to the B1 (CD20) antigen which is found specifically on B lymphocytes [5] and has been used to treat chronic lymphocytic leukemia or small lymphocytic lymphoma in first remission; and ^177^Lu-girentuximab, used in the treatment of metastatic clear cell renal cell carcinoma (ccRCC) [6]. Although several radionuclides have been used for labeling various (Mo)Abs, the *β*-emitters iodine-131 and yttrium-90 are the most commonly used radionuclides for clinical RIT. However, disadvantages in using iodine-131 for RIT include the metabolic lability of the labeled protein due to the deiodination and the *γ* emissions that account for two-thirds of its released energy causing an undesirable high absorbed dose to patients' bodies. Furthermore, the mean path length of the *β*^−^ particles is only about 0.44 mm (*E*_*β*,max_ = 0.606 keV), resulting in great heterogeneity of the radiation dose and little benefit from cross-fire. In contrast, yttrium-90 is a pure *β*-emitter, thus has fewer environmental radiation restrictions, and has higher energy (*E*_*β*,max_ = 2.28 MeV) and a particle range of up to 12 mm, making it more suitable for irradiation of larger tumors. On the other hand, yttrium-90 should be conjugated to (Mo)Ab via chelating agent, whereas iodine-131 can form a carbon-iodine bond directly.

Among alternative therapeutic radionuclides, rhenium-186 and rhenium-188 have desirable particulate emission characteristics for RIT [7] and are characterized by a very rich chemistry, typical of transition metals, which pave the way for different radiolabeling approaches.

Rhenium-186 (*t*_1/2_ = 90 h) with its 1074 keV *β*-emission and a 137 keV *γ*-emission, useful for imaging during the therapy, may be ideal from the viewpoint of physical properties. Unfortunately, the reactor produced rhenium-186 is a carrier added radionuclide with lower specific activity, due to the presence of cold rhenium-185 excess, compared to the generator produced rhenium-188 that is instead carrier-free and available from an in-house generator system similar to the technetium-99m one currently in wide use. The availability of carrier-free [^188^Re]ReO_4_^−^ by saline elution of a tungsten-188/rhenium-188 generator system provides rhenium-188 at any time in the clinical setting [8]. Furthermore, because of the high-energy *β*-particles, rhenium-188 (*E*_*β*max_ = 2.1 MeV) has a longer mean path length (about 2.2 mm) than iodine-131 or rhenium-186, which results in a more homogeneous distribution of the radiation dose.

The major concern for this isotope is its short half-life, 17 hours, which could be limiting with the maximum tumor uptake of radiolabeled antibodies. Quantitative measurements of the distribution of an iodine-131 labeled antibody to carcinoembryonic antigen carried out in relation to time by Begent et al. [9] have demonstrated that the maximum tumor uptake of radiolabeled IgG injected into humans occurs within 8 hours after administration. Moreover, Lucas et al. [10] and Xiao et al. [11] demonstrated that rhenium-188 is the best candidate for solid tumors treatment such as for non-small-cell lung cancer (NSCLC).

The aim of this review is to provide a short overview on the available labeling methods, quality controls, and in vitro stability of ^188^Re-labeled (Mo)Abs for radio immunotherapy. The methods reported in this paper can be conveniently applied for the labeling of (Mo)Ab with rhenium-188 for solid tumor, hematologic tumors, and epithelial cancers.

Even if we have attempted to be exhaustive, we apologize in advance for any inadvertent omissions.

## 2. Direct Radiolabeling of Antibodies with Generator Eluted [^188^Re]ReO_4_^−^

The most common approach utilized for the rhenium-188 antibodies labeling involves the reaction of the tetraoxo anion [^188^Re]ReO_4_^−^ with SnCl_2_, followed by the coordination of the reduced metal center by free thiol groups generated through the cleavage of protein's disulfide bridges (S-S). This simple method, known as “direct radiolabeling,” has been proved in the past to be a promising approach. In particular, Ferro-Flores et al. [12] and Griffiths et al. [13–15] demonstrated that this method could be easily adaptable to an instant “kit” for carrier-free rhenium-188 radiolabeling of antibodies and fragments, which retain their immunoreactivity. Later, several direct radiolabeling procedures have been developed making the use of ^188^Re-labeled antibodies achievable for research and clinical applications, thanks to the development of freeze-dry kit formulations [16–18]. Direct labeling procedures involve the prereduction of protein disulfide bridges, which can be carried out with different reducing agent such as 2-mercaptoethanol (2-ME) [12–15], stannous chloride (SnCl_2_) [16], ascorbic acid (AA) [19], tris-(2-carboxyethyl) phosphine (TCEP), and similar reducing agents [20–22] in order to generate sulfhydryl groups that can be conveniently used as coordinating sites to bind rhenium atoms in a reduced oxidation state. After the reduction of the protein's disulfide bridges S-S and before the labeling with rhenium-188, if 2-ME, TCEP, and other reagents, able to coordinate the nuclide rhenium-188, are used as reductant, it is necessary to purify the reduced antibody (or the antibody fragment) from excess of the reducing agents, which could compete for the coordination to the metal center subtracting it from the reaction environment. To achieve this aim size exclusion chromatography is generally used. The solution containing the reduced antibody is loaded onto a size exclusion column; the antibody is collected in the first eluting fractions leaving into the column the smaller size molecules as TCEP, 2-ME, and so on. The protein concentration is generally calculated using UV-visible spectrometry after treatment with protein assay kits and the free-SH per (Mo)Abs number is determined using Ellman's reaction by reference to a standard curve.

**Figure 1:**
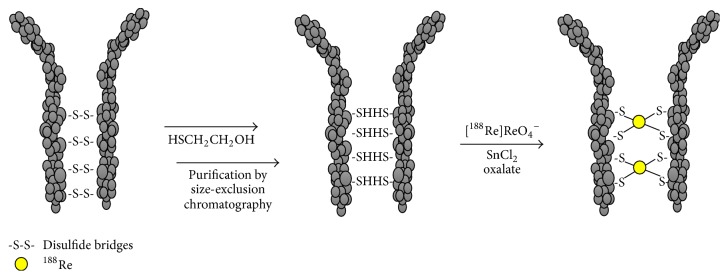
Schematic representation of the direct radiolabeling of antibodies with generator eluted [^188^Re]ReO_4_^−^, using oxalate procedure.

The reduced-purified antibodies (Mo)Abs are then incubated with the generator eluted [^188^Re]ReO_4_^−^ in the presence of a reducing agent (SnCl_2_); the radiolabeling should be performed under acidic condition (pH 4.5–5) to avoid the formation of the insoluble reduced specie [^188^Re]ReO_2_ [23]. Generally, this procedure requires the use of high amount of tin and long incubation time, due to the critical reduction step of [^188^Re]ReO_4_^−^ [24]. It was found that the addition of sodium oxalate to the reaction mixture dramatically decreases the time required to obtain a satisfactory yield of rhenium-188 incorporation into the antibodies' structure, suggesting that the reduction of perrhenate anion has to be considered as the limiting step in the labeling procedure. The authors assumed that the presence of oxalate (Figure 1) promotes the reduction of the metal and the coordination by the free thiol sulfur groups of the reduced antibody took place quite rapidly giving rise to a stable incorporation of the nuclide into the humanized monoclonal antibody h-R3 [24]. Also Kothari et al. [18] demonstrated that the addition of sodium oxalate to the ^188^Re labeling of the reduced monoclonal antibody CAMA3C8, specific for breast cancer, helps to increase the reduction ability and hence labeling yield [18]. These results are in close agreement with data obtained in applying the same approach to the preparation of ^188^Re-labeled radiopharmaceuticals [25, 26].

Recently, new approaches have been developed for labeling bioactive molecules [27–29], such as antibodies and fragments with rhenium-188. A promising and suitable strategy for the labeling of antibodies with rhenium-188 was proposed by Dias et al. [30]. In this study they presented an alternative direct labeling approach based on the reaction of the [^188^Re][Re(CO)_3_]^+^ with the reduced anti-CD20 antibody rituximab as potential alternative to the ^90^Y-Zevalin for therapy of non-Hodgkin's lymphoma, demonstrating that the use of the tricarbonyl core can be a promising and suitable strategy for rhenium-188 antibodies labeling. The precursor [^188^Re][Re(CO)_3_]^+^ was prepared through a two-step kit preparation. In first step the generator eluted [^188^Re]ReO_4_^−^ was mixed with HCl (4 M), 2-[morpholino]ethanesulfonic acid buffer (0.5 M) and ascorbic acid. The resulting mixture was purged with argon and made to react with BH_3_NH_3_ and flushed with CO before incubating at 80°C for 1 h [30].

## 3. Indirect Radiolabeling of Antibodies with Generator Eluted [^188^Re]ReO_4_^−^

Direct approaches for the labeling of antibodies with ^188^Re are simple and efficient; however, they suffer from being site-unspecific and in some case the final label results are unstable [23].

In the past, prelabeling (preconjugation) and postlabeling (postconjugation) approaches have been developed as alternative approaches to direct labeling of antibodies with radiorhenium. The main difference between the two strategies concerns the use in the indirect labeling of an endogenous bifunctional chelating agent (BCA). A BCA is a particular chemical moiety characterized by two essential functions: a part of the molecule must be able to bind the biologically active molecule and the other to firmly chelate the radiometal. Specific exogenous chelators must be chemically modified in order to possess both conjugations and chelating functions [31]. Indirect labeling can be site-specific and versatile but is more complicated than the direct one.

**Figure 2:**
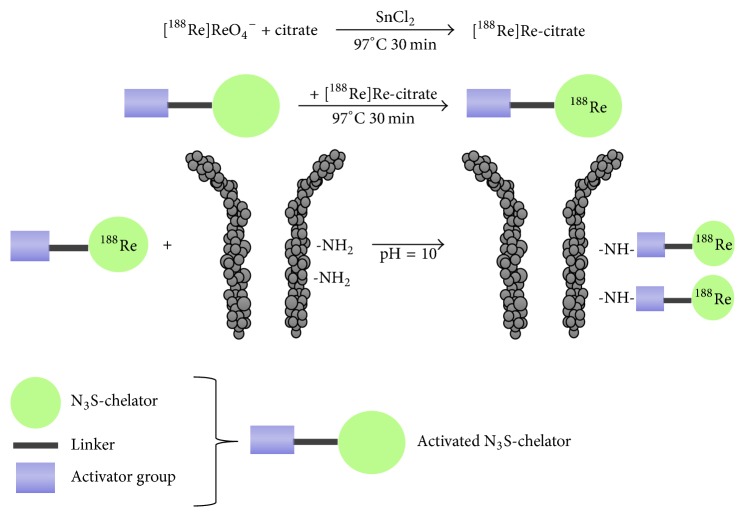
Schematic representation of indirect radiolabeling of antibodies with generator eluted [^188^Re]ReO_4_^−^ using the prelabeling approach. An activated N_3_S-chelator is first labeled with rhenium-188 by traschelation of a first produced [^188^Re]Re-citrate intermediate complex and then the resulting compound is conjugated to the antibody [32–34].

**Figure 3:**
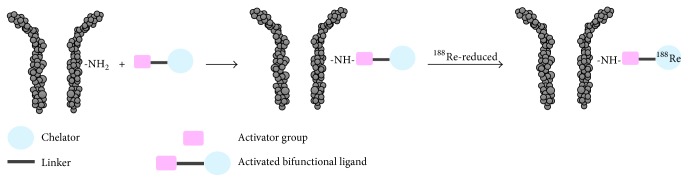
Schematic representation of the indirect postconjugation approach.

In the case of prelabeling approach, an activated BCA is labeled with radiorhenium, before conjugating to the antibody (Figure 2). In this example an activated N_3_S-chelator is first labeled with rhenium-188 by traschelation of a previously produced ^188^Re-citrate intermediate complex and then the resulting compound is conjugated to the antibody. As shown in Figure 2, the radiolabeling of the N_3_S-chelator was performed by heating at high temperature for 30 minutes in the presence of stannous citrate [32–34]. One disadvantage of preconjugation labeling concerns the need of postlabeling purification. On the other hand, the previous reaction scheme is not suitable for postconjugation labeling, since subjecting an antibody to high temperature for long time and with high concentration of tin ions would denature the protein. For this reason, few examples of postconjugation radiorhenium are reported up to now [35–38]. In the postconjugation radiolabeling techniques, a BCA serves as a linker between the antibody and the radiometal, the BCA is first conjugated to the antibody, and the resulting conjugate is then radiolabeled in a second step (Figure 3). Among the few examples, relevant is the use of the trihydroxamate BCA, trisuccin, as potential ligand for radiolabeling of (Mo)Abs with radiorhenium, through an indirect postconjugation approach [39]. Indeed, Safavy et al. [39] demonstrated that the use of this trihydroxamate BCA made it possible to prepare stable BCA-(Mo)Ab conjugates in pure form that can be radiolabeled with carrier-free rhenium-188. Trisuccin-(Mo)Ab conjugates were synthesized at different BCA: (Mo)Ab ratios by the 6-oxoheptanoic acid method using trisuccin hydrazide [39] and radiolabeled by incubation with the prereduced rhenium-188 at 45°C for 45 min. The authors proved that the method efficiently produces ^188^Re-hydroxamate-antibody conjugates in high yield and with high stability in vivo. In Figure 3 a schematic representation of the indirect postconjugation approach is reported.

## 4. Determination of the ^188^Re-Radioimmunoconjugates Radiochemical Purity

After labeling of (Mo)Abs with rhenium-188, three main radioactive species can be present in the labeling solution: the unreacted [^188^Re]ReO_4_^−^; the hydrolyzed reduced [^188^Re]ReO_2_; and of course the ^188^Re-(Mo)Ab [22, 24]. Radiochemical purity is in general determined by ascending chromatography. Different combined procedures can be applied in order to separate these radioactive compounds [24]: (1) human serum albumin (HSA 1%) impregnates ITLC-SG, or TLC-SG strips are used as stationary phase and ammonium hydroxide : ethanol : water (1 : 2 : 5) as mobile phase to separate radiocolloids [^188^Re]ReO_2_, which remain at the bottom, while the radiolabeled (Mo)Abs and free perrhenate moved to the top; (2) silica gel plates using methanol : water (85 : 15) as mobile phase are used to separate radioactive colloids and the labeled antibody, which remain at the bottom, while free perrhenate migrates to the top; (3) Whatman paper developed with acetone to separate the hydrolyzed reduced [^188^Re]ReO_2_ and ^188^Re-labeled (Mo)Ab, which remain at the bottom, while free perrhenate migrates to the top. Relative front (*R*_*f*_) values of ^188^Re-radiochemical species running in different solvent systems are reported in Table 1.

Another method for the radiochemical purity determination of radiolabeled antibodies is the gel chromatography. Generally, the sample is loaded on a size exclusion Sephadex G-25 column equilibrated by nitrogen purged phosphate buffer (pH 7.5) and eluted using the same buffer [18]. One mL fraction has to be collected and counted using a radiometric detector (e.g., NaI(Tl) scintillation detector). Using this method, the radiolabeled antibody is collected in the first 5–7 ml, while the smaller size ^188^Re-species, such as free perrhenate and radiocolloids, are more retained on the column and collected after 15–20 mL of buffer.

On the same principle base of chromatography, size exclusion radio-HPLC analysis can be performed to determine the radiochemical purity of the final ^188^Re-labeled (Mo)Ab. Radiochemical species can be separated according to the molecular size and weight, and the radiolabeled antibody can be resolved form size smaller ^188^Re-species. Seitz et al. [20] used radio-HPLC to measure the purity of the ^188^Re-labeled anti-NCA antigen (nonspecific cross-reacting antigen) (Mo)Ab BW 250/183. Radio-HPLC analysis was performed on a 300 × 7.8 mm Bio-Sil SEC 250–5 size exclusion column with a flow rate of 1 mL/min, equilibrated with 0.1 M sodium phosphate buffer, pH 6.75, containing 0.01 M sodium azide. In this system the ^188^Re-labeled (Mo)Ab BW 250/183 shows a retention time of 9.4 min, while free perrhenate is eluted after 15 min.

Size exclusion chromatography can be also used to investigate the integrity of ^188^Re-labeled (Mo)Ab, for in vitro stability tests, and to purify the radiolabeled antibodies from excess of reagents and by-products [13].

## 5. Stability Studies of ^188^Re-Radioimmunoconjugates

To test the stability of ^188^Re-antibodies after radiolabeling, the radioimmunoconjugates are firstly purified by size exclusion chromatography and then an aliquot of the purified rhenium compound incubated in 5% HSA at 37°C for a time from 24 to 120 h [13, 18, 20]. The radiochemical purity is estimated by paper, TLC, or gel radiochromatography. The stability can be also checked by incubation in saline and serum. In general, ^188^Re-radioimmunoconjugates are found to be stable until 24 h and loss of radioactivity appears as free perrhenate when the direct labeling procedure is used. In these cases, the addition of gentisic acid slows down the degradation and increases the stability in vitro [20].

## 6. Conclusion

The purpose of this paper was to give a general update on the available procedure to label (Mo)Abs with rhenium-188 and how to check their radiolabeling yield and stability in vitro. In fact, rhenium-188 is actually considered as one of the most promising *β*-emitting radionuclide candidates for therapeutic applications. It is easily available through a tungsten-188/rhenium-188 generator system, similar to the molybdenum-99/technetium-99m generator, and could be obtained to the need by elution straight in radiopharmacy. A further advantage is due to the *γ*-emission associated with its decay that can be conveniently used for imaging studies and dosimetry. Furthermore, the similarities between the congener technetium elements have allowed transferring all the knowledge about the preparation of the ^99m^Tc-labeled radiopharmaceuticals on the analogues ^188^Re-labeled radiopharmaceuticals. The rhenium-188 direct labeling to (Mo)Abs was carried out with success starting from the known labeling strategies for the technetium-99m antibodies labeled adapted to rhenium-188. Despite the fact that the two elements are characterized by similar chemical properties they differ in the value of the standard reduction potential of their tetraoxo anions MO_4_^−^ (*M* = Tc, Re), which is lower for perrhenate than for pertechnetate. Hence, in general the preparation of ^188^Re-radiopharmaceuticals needs more drastic reaction condition compered to ^99m^Tc-radiopharmaceuticals preparation, such as high amount of tin, high incubation temperature, higher ligand concentration, and very acidic condition, all conditions not compatible with the integrity of the antibodies. In the last years, it has been reported that the use of oxalate ions favors the reduction of [^188^Re]ReO_4_^−^ possibly due to the formation of an intermediate Re(VII) complex with oxalate [18, 24] and this dramatically decreases the time required to obtain satisfactory ^188^Re-labeled (Mo)Abs yield and the amount of tin and allows carrying out the labeling at not too acidic pH.

Although promising alternative approaches are available for Re-188 antibodies radiolabeling, such as prelabeling and postlabeling methods, the direct approach still remains a convenient and efficient procedure (Table 2) that avoids the use of bifunctional chelating agents.

## Figures and Tables

**Table 1:** *R*_*f*_ values of ^188^Re-radiochemical species running in different solvent systems.

Stationary phase	Mobile phase	^188^Re-species	*R*_*f*_
ITLC-SG	NH_4_OH : EtOH : H_2_O (1 : 2 : 5)	[^188^Re]ReO_2_	0
		[^188^Re]ReO_4_^−^	1
		^188^Re-MoAb	1
	MeOH : H_2_O (85 : 15)	[^188^Re]ReO_2_	0
		[^188^Re]ReO_4_^−^	1
		^188^Re-MoAb	0
	NaCl 0.9%	[^188^Re]ReO_2_	0
		[^188^Re]ReO_4_^−^	1
		^188^Re-MoAb	0
	Acetone	[^188^Re]ReO_2_	0
		[^188^Re]ReO_4_^−^	1
		^188^Re-MoAb	0

Whatman paper	Acetone	[^188^Re]ReO_2_	0
		[^188^Re]ReO_4_^−^	0.7–1
		^188^Re-MoAb	0
	NaCl 0.9%	[^188^Re]ReO_2_	0
		[^188^Re]ReO_4_^−^	0.6–1
		^188^Re-MoAb	0

**Table 2:** Pros and cons between direct and indirect labeling methods.

Labeling methods	Pros	Cons
Direct	(i) Simpler approach (ii) Less complicated antibody preparation (iii) Fewer reaction steps (iv) Reactions frequently conducted at room temperature (v) Rapid procedure (vi) More appropriate for freeze-dried kit formulation	(i) Site unspecific (ii) Less stable radioimmunoconjugates

Indirect	(i) Site specific (ii) More stable radioimmunoconjugates	(i) More complicated approach (ii) More complicated antibody preparation (iii) More reaction steps (iv) Reactions frequently conducted at high temperature (v) Slow procedure (vi) Postradiolabeling purification
